# Supplementary material for: Identification and functional analysis of non-coding regulatory small RNA FenSr3 in Bacillus amyloliquefaciens LPB-18
Source: PeerJ. 2023 May 15;11:e15236. doi: 10.7717/peerj.15236 (PMC10194069; doi:10.7717/peerj.15236)
Supplement: Supplemental Information 4 [file peerj-11-15236-s004.zip › KO/CK-vs-T1_map/map04623.html]

KEGG PATHWAY: Cytosolic DNA-sensing pathway - Reference pathway


|  |  |
| --- | --- |
| **Cytosolic DNA-sensing pathway - Reference pathway** |  |

[
Pathway menu
| Organism menu
| Pathway entry
| Show description
| User data mapping
]

Specific families of pattern recognition receptors are responsible for detecting foreign DNA from invading microbes or host cells and generating innate immune responses. DAI is the first identified sensor of cytosolic DNA which activates the IRF and NF-{kappa}B transcription factors, leading to production of type I interferon and other cytokines. The second type of cytoplasmic DNA sensor is AIM2. Upon sensing DNA, AIM2 triggers the assembly of the inflammasome, culminating in interleukin maturation. In addition to these receptors, there is a mechanism to sense foreign DNA, with the host RNA polymerase III converting the DNA into RNA for recognition by the RNA sensor RIG-I. These pathways provide various means to alert the cell.

|  |  |  |
| --- | --- | --- |
| Reference pathway | 184% 150% 122% 100% 82% 67% 55% | 图片下载 |
